# Multidrug-resistant *Klebsiella pneumoniae* harboring extended spectrum β-lactamase encoding genes isolated from human septicemias

**DOI:** 10.1371/journal.pone.0250525

**Published:** 2021-05-04

**Authors:** Isabel Carvalho, Nadia Safia Chenouf, José António Carvalho, Ana Paula Castro, Vanessa Silva, Rosa Capita, Carlos Alonso-Calleja, Maria de Lurdes Nunes Enes Dapkevicius, Gilberto Igrejas, Carmen Torres, Patrícia Poeta

**Affiliations:** 1 Department of Veterinary Sciences, Microbiology and Antibiotic Resistance Team (MicroART), University of Trás‐os‐Montes and Alto Douro, Vila Real, Portugal; 2 Department of Genetics and Biotechnology, UTAD, Vila Real, Portugal; 3 Functional Genomics and Proteomics Unit, UTAD, Vila Real, Portugal; 4 Laboratory Associated for Green Chemistry (LAQV‐REQUIMTE), New University of Lisbon, Monte da Caparica, Portugal; 5 Area Biochemistry and Molecular Biology, University of La Rioja, Logroño, Spain; 6 Medical Center of Trás-os-Montes e Alto Douro E.P.E., Vila Real, Portugal; 7 Department of Food Hygiene and Technology, Veterinary Faculty, University of León, León, Spain; 8 Institute of Food Science and Technology, University of León, León, Spain; 9 University of the Azores, Faculty of Agricultural and Environmental Sciences (M.L.E.D.), Angra do Heroísmo, Portugal; 10 Institute of Agricultural and Environmental Research and Technology (IITAA), University of the Azores, Angra do Heroísmo, Portugal; Nitte University, INDIA

## Abstract

*Klebsiella pneumoniae* is a major pathogen implicated in nosocomial infections. Extended-spectrum β-lactamase (ESBL)-producing *K*. *pneumoniae* isolates are a public health concern. We aim to characterize the type of β-lactamases and the associated resistance mechanisms in ESBL-producing *K*. *pneumoniae* isolates obtained from blood cultures in a Portuguese hospital, as well as to determine the circulating clones. Twenty-two cefotaxime/ceftazidime-resistant (CTX/CAZ^R^) *K*. *pneumoniae* isolates were included in the study. Identification was performed by MALDI-TOF MS and the antimicrobial susceptibility testing by disk-diffusion. The screening test for ESBL-production was performed and ESBL-producer isolates were further characterized. The presence of different beta-lactamase genes (*bla*_CTX-M_, *bla*_SHV_, *bla*_TEM_, *bla*_KPC_, *bla*_NDM,_ *bla*_VIM,_ *bla*_OXA-48,_ *bla*_*CMY-2*_, *bla*_DHA-1,_ *bla*_FOX,_ *bla*_MOX,_ and *bla*_ACC_) was analyzed by PCR/sequencing in ESBL-producer isolates, as well as the presence of other resistance genes (*aac(6’)-Ib-cr*, *tet*A/B, *dfrA*, *qnr*A/B/S, *sul*1/2/3) or integron-related genes (*int*1/2/3). Multilocus-sequence-typing (MLST) was performed for selected isolates. ESBL activity was detected in 12 of the 22 CTX/CAZ^R^ *K*. *pneumoniae* isolates and 11 of them carried the *bla*_CTX-M-15_ gene (together with *bla*_TEM_)_,_ and the remaining isolate carried the *bla*_SHV-106_ gene. All the *bla*_CTX-M-15_ harboring isolates also contained a *bla*_SHV_ gene (*bla*_SHV-1_, *bla*_SHV-11_ or *bla*_SHV-27_ variants). Both *bla*_SHV-27_ and *bla*_SHV-106_ genes correspond to ESBL-variants. Two of the CTX-M-15 producing isolates carried a carbapenemase gene (*bla*_KPC2/3_ and *bla*_OXA-48_) and showed imipenem resistance. The majority of the ESBL-producing isolates carried the *int*1 gene, as well as sulphonamide-resistance genes (*sul*2 and/or *sul*3); the *tetA* gene was detected in all eight tetracycline-resistant isolates. Three different genetic lineages were found in selected isolates: ST348 (one CTX-M-15/TEM/SHV-27/KPC-2/3-producer isolate), ST11 (two CTX-M-15/TEM/SHV-1- and CTX-M-15-TEM-SHV-11-OXA-48-producer isolates) and ST15 (one SHV-106/TEM-producer isolate). ESBL enzymes of CTX-M-15 or SHV-type are detected among blood *K*. *pneumoniae* isolates, in some cases in association with carbapenemases of KPC or OXA-48 type.

## 1. Introduction

During the last decades, the selective pressure exerted by antibiotics has given rise to bacterial species which are increasingly resistant to these agents, and this increase in multi-resistant pathogenic strains has been extremely high [1, 2].

*Klebsiella pneumoniae* is a major pathogen implicated in nosocomial infections that is known to spread easily, and it is frequently associated with resistance to the highest-priority critically important antimicrobial agents [3, 4]. During the last years, the diffusion of broad-spectrum cephalosporin-, carbapenem- and colistin-resistant *K*. *pneumoniae* isolates is now reducing treatment options and the containment of infections. Recently, the World Health Organization (WHO) [5] published a global priority list of antibiotic resistant bacteria, where third-generation cephalosporin- and/or carbapenem-resistant *Enterobacteriaceae* (*K*. *pneumoniae* and others), were included in the Priority 1 group. According to Amit, Mishali [6], carbapenem-resistant *K*. *pneumoniae* isolates implicated in bloodstream infections are associated with a high mortality rate of 40% to 70%.

*K*. *pneumoniae* isolates can acquire different mechanisms that confer antibiotic resistance to commonly used antibiotics. Among the most common mechanisms, the Extended-spectrum β-lactamases (ESBLs) and Acquired AmpC enzymes (qAmpCs) are widely reported [6–8]. One of the main concerns is that resistance caused by these enzymes may result in efficacy reduction of antimicrobial therapy, or in failed treatment [9]. Carbapenems are considered a last-resort antibiotic group for the treatment of infections caused by multidrug-resistant (MDR) *Enterobacteriaceae* [10]. ESBL- and carbapenemase-producing *K*. *pneumoniae* isolates are usually found after prolonged hospital stay and tends to affect debilitated patients with poor functional status [11]. Antimicrobial resistance is commonly related to the spread of plasmids, and the acquisition of resistance genes that normally occur by horizontal gene transfer (HGT) [12, 13]. International high-risk clones of *K*. *pneumoniae* are frequently detected not only among humans’ infections but also in those of companion animals [14–21].

Previous studies have been performed in Portugal analyzing the diversity of ESBLs in clinical *K*. *pneumoniae* isolates [4, 22–26], but none of them have been performed in our geographical region among invasive infections. The aim of this study was to characterize the type of ESBLs and the associated resistance mechanisms in broad-spectrum cephalosporin-resistant *K*. *pneumoniae* isolates recovered from blood cultures in a Portuguese hospital, as well as to determine the genetic lineages of these isolates.

## 2. Materials and methods

### 2.1 Bacterial isolates

A collection of 22 cefotaxime/ceftazidime-resistant (CTX/CAZ^R^) *K*. *pneumoniae* isolates obtained from blood cultures of hospitalized patients (one isolate/patient) in a Portuguese hospital (*Centro Hospitalar de Trás os Montes e Alto Douro*, CHTMAD) between january 2017 and september 2018, were included in this study. Identification was confirmed by matrix-assisted laser desorption/ionization time-of-flight mass spectrometry method (MALDI-TOF MS, Bruker).

### 2.2 Susceptibility testing

Antimicrobial susceptibility testing was performed by Kirby–Bauer disk diffusion method on Mueller-Hinton agar, according with Clinical Laboratory Standards Institute guidelines (CLSI, 2019) [27]. The susceptibility of *K*. *pneumoniae* isolates was tested for the following antibiotics (μg/disk): amoxicillin + clavulanic acid (20+10), cefoxitin (30), ceftazidime (30), cefotaxime (30), imipenem (10), tetracycline (30), gentamicin (10), streptomycin (10), tobramycin (10), ciprofloxacin (5) and trimethoprim-sulfamethoxazole (SXT, 1.25+23.75). The screening of phenotypic ESBL production was carried out by the double disk synergy test using cefotaxime, ceftazidime and amoxicillin/clavulanic acid discs [27]. Isolates showing a positive ESBL-screening test were selected for further characterization in this study.

### 2.3 DNA extraction and quantification

Genomic DNA from ESBL-producing *K*. *pneumoniae* isolates was extracted using the InstaGene Matrix (Bio-Rad), according to the manufacturer’s instructions.

### 2.4 Antibiotic resistance genes

PCR (polymerase chain reaction) was the selected methodology to analyze the presence of resistance genes. *K*. *pneumoniae* isolates were screened by PCR and sequencing for the presence of genes encoding beta-lactamases: *bla*_CTX-M_, *bla*_SHV_, *bla*_TEM_, *bla*_CMY-2_, *bla*_DHA-1,_ *bla*_FOX,_ *bla*_MOX,_ *bla*_ACC,_ *bla*_KPC_, *bla*_OXA-48_, *bla*_VIM_ and *bla*_NDM_ [28, 29]. The isolates were also screened by PCR (and sequencing when required) for the presence of the genes encoding for resistance to tetracycline (*tet*A, *tet*B), fluoroquinolones (*aac(6´)-Ib-cr*, *qnrA*, *qnrB*, and *qnrS*), sulfamethoxazole (*sul1*, *sul2* and *sul3*), and trimethoprim (*dfrA* genes) [28]. The presence of the integrase gene of class 1, class 2 and class 3 integrons (*int1*, *int2* and *int3*, respectively) were analyzed by PCR [30]. Furthermore, the *mcr*-1 colistin resistance gene was tested in all *K*. *pneumoniae* isolates [31]. Analysis of DNA sequences was performed with the BLAST program, available at the National Center for Biotechnology Information. Positive controls of the University of La Rioja were used in all PCR assays.

### 2.5 Multilocus sequence typing of K. pneumoniae isolates

The multilocus-sequence-typing (MLST) with seven housekeeping genes (*gap*A, *pho*E, *inf*B, *pgi*, *rpo*B, *ton*B and *mdh*) was performed by PCR and sequencing in selected *K*. *pneumoniae* isolates (https://bigsdb.pasteur.fr/klebsiella/klebsiella.html); the allelic combination of the seven genes allowed the determination of the sequence type (ST).

## 3. Results

### 3.1 Antimicrobial resistance phenotype in CTX/CAZR K. pneumoniae isolates

Amongst the 22 CTX/CAZ^R^ *K*. *pneumoniae* isolates, twelve of them showed a positive ESBL screening test (54.5%) and these isolates were considered for further genetic resistance analysis.

Considering the 12 ESBL-positive isolates, different levels of resistance were recorded towards amoxicillin+clavulanic acid, trimethoprim/sulfamethoxazole and ciprofloxacin (100%), tobramycin (91.7%), gentamicin (75%), tetracycline (66.7%), streptomycin (41.7%) or cefoxitin (25%). Accordingly, all these *K*. *pneumoniae* isolates showed a MDR-phenotype.

### 3.2 Genetic determinants in ESBL-producing K. pneumoniae isolates

As shown in **Table 1**, most of the ESBL-producing *K*. *pneumoniae* isolates (11 out of 12) carried the *bla*_CTX-M-15_ gene and co-harbored a β-lactamase gene of SHV-type, with the following variants (number of isolates): *bla*_SHV-1_ (9 isolates), *bla*_SHV-11_ (1 isolate) and *bla*_SHV-27_ (1 isolate, ESBL-variant). Moreover, the remaining ESBL-producing isolate carried the *bla*_SHV-106_ gene, a genetic variant that confers an ESBL phenotype. A *bla*_TEM_ gene was found among most of the ESBL-producing isolates (all except one). Three of the ESBL-positive isolates showed resistance to cefoxitin and amoxicillin-clavulanic acid, but all were negative by PCR for the genes encoding qAmpC beta-lactamases (*bla*_CMY-2_, *bla*_DHA-1,_ *bla*_FOX,_ *bla*_MOX,_ and *bla*_ACC_). Moreover, four of the ESBL-positive isolates also were IMP^R^; a carbapenemase gene was detected in two of these isolates: 1) one of them carried the *bla*_KPC2/3_ gene (it was not possible to distinguish between both variants after sequencing the PCR amplicon), together with *bla*_CTXM-15_, *bla*_SHV-27_ and *bla*_TEM_ genes; 2) the other one carried the *bla*_OXA-48_ gene, together with *bla*_CTXM-15_, *bla*_SHV-11_ and *bla*_TEM_ genes (**Table 1**); the two remaining IMP^R^ isolates were negative for all carbapenemase genes tested.

**Table 1:** Resistance phenotype and genotype present in *K*. *pneumoniae* isolates from human septicemias in Portugal.

Samples	Resistance phenotype^a^	ESBL production^b^	Beta-lactamases	MLST^c^	Other genes	Integrase
X1089	AMC, CAZ, CTX, IMP, CIP, SXT, TET, S	P	CTX-M-15, TEM, SHV-11, OXA-48	ST11	*tetA*, *sul2*, *aac(6¨)-Ib-cr*	*int1*
X1090	AMC, FOX, CAZ, CTX, IMP, CIP, SXT, TET, GN, S, TOB	P	CTX-M-15, TEM, SHV-27, KPC2/3	ST348	*tetA*, *sul2*, *aac(6¨)-Ib-cr*, *qnrS*	*int1*
X1091	AMC, FOX, CAZ, CTX, IMP, CIP, SXT, TET, GN, S, TOB	P	CTX-M-15, TEM, SHV-1		*tetA*, *sul2*, *aac(6¨)-Ib-cr*, *qnrS*	*int1*
X1096	AMC, CAZ, CTX, CIP, SXT, TET, GN, TOB	P	CTX-M-15, TEM, SHV-1		*tetA*, *sul2*, *qnrS*	*int1*
X1097	AMC, CAZ, CTX, CIP, SXT, TET, TOB	P	CTX-M-15, TEM, SHV-1	ST11	*tetA*, *sul2*	*int1*
X1098	AMC, CAZ, CTX, CIP, SXT, TET, GN, TOB	P	CTX-M-15, TEM, SHV-1		*tetA*, *sul2*, *qnrS*	*int1*
X1099	AMC, CAZ, CTX, CIP, SXT, TET, GN, TOB	P	CTX-M-15, SHV-1		*tetA*, *qnrS*	*int1*
X1100	AMC, CAZ, CTX, CIP, SXT, GN, TOB	P	CTX-M-15, SHV-1, TEM		*sul2*, *sul3*, *qnrS*	*int1*
X1101	AMC, CAZ, CTX, CIP, SXT, GN, TOB	P	CTX-M-15, SHV-1, TEM		*sul2*, *sul3*, *qnrS*	*int1*
X1102	AMC, CAZ, CTX, CIP, SXT, GN, TOB	P	CTX-M-15, SHV-1, TEM		*sul2*, *sul3*, *qnrS*	*int1*
X1103	AMC, CAZ, CTX, CIP, SXT, TET, GN, S, TOB	P	CTX-M-15, SHV-1, TEM		*tetA*, *sul2*, *sul3*, *qnrS*	*int1*
X1088	AMC, FOX, CAZ, CTX, IMP, CIP, SXT, S, TOB	P	SHV-106, TEM	ST15	*sul2*, *sul3*, *aac(6¨)-Ib-cr*, *qnrS*	

^**a**^AMC: amoxicillin+clavulanic acid; FOX: cefoxitin; CTX: cefotaxime; CAZ: ceftazidime; ATM: aztreonam; IMP: imipenem; TET: tetracycline; CIP: ciprofloxacin; SXT: trimethoprim+sulfamethoxazole; GN: gentamicin; TOB: tobramycin; S: streptomycin; FOX: cefoxitin.

^**b**^P–Positive, N- Negative.

^c^MLST–MultiLocus Sequence Typing.

Tetracycline resistance was mediated in all eight resistant isolates by the *tet*A gene and the *int*1 gene, encoding the integrase of class 1 integrons, was present in 11 out of 12 ESBL-producing isolates (**Table 1).** The *int2* and *int3* genes (encoding the integrase of class 2 and 3 integrons, respectively) were not detected in this study. Furthermore, sulfamethoxazole-resistance was mediated by *sul*2 (n = 10) and *sul*3 genes (n = 5) in ESBL-producers. The *aac(6¨)-Ib-cr* gene was detected in four ciprofloxacin-resistant isolates and *qnr*S was identified among 10 isolates (**Table 1**).

MLST analysis was performed in four representative *K*. *pneumoniae* isolates (based on the antimicrobial resistance genotype), and revealed three different lineages: ST348 (in one ESBL and IMP^R^ isolate carrying the genes encoding CTX-M-15, TEM, SHV-27, and KPC2/3 enzymes), ST11 (in two isolates, one of them carried CTX-M-15+TEM+SHV-1 and the other CTX-M-15+TEM+SHV-11+OXA-48) and ST15 (in one ESBL-positive isolate with SHV-106+TEM) (**Table 1**).

## 4. Discussion

The mechanisms of resistance implicated in a collection of ESBL-producing *K*. *pneumoniae* isolates obtained from invasive infections (blood cultures) in a Portuguese hospital have been analyzed in this study. In agreement with the current global epidemiology based on the *bla*_CTX-M_, we detected the CTX-M-15 β-lactamase in most of ESBL-producer isolates (11 out of 12). This enzyme is widely disseminated among human isolates, particularly in Portugal [8, 23–26, 32]. It is worth noting that the first report of the CTX-M-15 enzyme isolated from blood culture in Portuguese hospitals goes back to 2005 [33]. Since then, the occurrence of this genotype was announced from *K*. *pneumoniae* isolates of various environments in Portugal, more recently in sick and healthy dogs [20], which can be explained by the close contact between humans and pets. Actually, it has been claimed that the *bla*_CTX-M-15_ among humans has, outstandingly, increased over time in most countries. Our finding seems to match completely with surveys conducted on hospitals located in different parts of Europe [19, 34–36]. Likewise, it was shown that this genotype has been disseminated in Asia [37] and Africa [38]. This study constitutes additional evidence that the CTX-M-15 remains the most important CTX-M enzyme in *K*. *pneumoniae* due to its large diffusion and relation to infections in human settings. Accordingly, this global spread could be, mostly, explained by the HGT between bacteria, mediated by conjugative plasmids [24]. Other ESBL variants of SHV-type were detected, either associated (SHV-27) or not associated to CTX-M-15 (SHV-106). Similarly, other authors detected SHV-106 producing *K*. *pneumoniae* isolates in Portuguese health institutions [24, 25, 39]. Moreover, SHV-27-producing *K*. *pneumoniae* isolates have been reported among human clinical infections [40, 41] and also in companion animals in Japan and Germany [42, 43]. This ESBL gene is frequently associated to other ESBL genes of the CTX-M-type, both in human and in animal settings.

Interestingly, two of the ESBL-producing isolates (with the *bla*_CTX-M-15_ gene, associated or not to *bla*_SHV-27_) also carried a carbapenemase encoding gene (*bla*_KPC2/3_ or *bla*_OXA-48_) as well as other beta-lactamase genes (*bla*_TEM_ and *bla*_SHV-11_). In this respect, the *bla*_OXA-48_ gene was found in one ESBL-producer isolate (recovered in 2017), in association with *bla*_CTX-M-15,_ *bla*_TEM_ and *bla*_SHV-11_ genes. Moreover, another isolate carried the *bla*_KPC2/3_ gene, together with two ESBL encoding genes (*bla*_CTX-M-15_ and *bla*_SHV-27_). Similarly, some international reports showed the presence of *bla*_OXA-48_ gene among hospitalized patients [44]. This gene had been reported in human isolates, mainly in Iberian Peninsula [45–48]. Particularly in Portugal, the OXA-181 carbapenemase was detected among *K*. *pneumoniae* isolates of hospitalized patients [8].

Moreover, carbapenemases of the KPC-2, KPC-3 and OXA-48 type have been recently reported among carbapenem resistant *K*. *pneumoniae* isolates of different origins from the same hospital analyzed in our study, few of them of blood origin [46, 49]. Carbapenems are generally considered the most effective antibacterial agents and the first-choice treatment for infections caused by ESBL-producing *Enterobacteriaceae*. The current study emphasizes the relevance of co-occurrence of ESBL and carbapenemase encoding genes in *K*. *pneumoniae* isolates implicated in invasive infections with the difficulties that could have for effective therapeutic options.

Three different sequence types belonging to major international high-risk *K*. *pneumoniae* clones were identified in this study among four selected *K*. *pneumoniae* producer isolates (ST11, ST15 and ST348), revealing clonal diversity, in line with previous reports [3, 14]. The ST11 lineage has been frequently detected worldwide among CTX-M-15- [50] and KPC-producing *K*. *pneumoniae* isolates [51]. In addition, isolates of ST348 or ST15 lineages producing CTX-M-15 and/or KPC-2/3 enzymes have been reported either in humans or animals in different studies performed in Portugal [21, 32, 46]. The ST15 lineage was identified in our study in a SHV-106-producing *K*. *pneumoniae* isolate, and similar isolates were previously circulating in a Portuguese hospital [24].

Rodrigues, *et al*. [24] considered that the dissemination of CTX-M-15 and the persistence of diverse ESBLs of SHV-type among *K*. *pneumoniae* isolates was mainly linked to a few epidemic and international clones (as ST15), in line with our study. It is important to note that this ST15 lineage has been disseminated in different settings. The ST15 lineage was found in an intensive care unit in Brazil [12] and among CAZ^R^ clinical isolates in France, Poland and Portugal [52]. Moreover, ertapenem-resistance associated with ST11, ST15 and ST348 *K*. *pneumoniae* lineages have been previously found in another hospital in the same region [32]. *K*. *pneumoniae* ST15, which is a high-risk clonal lineage, seems to predominate among clinical CTX-M-15-producing isolates from companion animals [3, 42, 53]. All these findings indicate that these clonal lineages are frequently circulating, suggesting their important contribution to the expansion of β-lactamases in Portuguese hospitals.

## 5. Conclusions

Antimicrobial resistance can make infections difficult to treat, which represents a public health problem due to the negative consequences for human health.

*Enterobacteriaceae* isolated from septicemias in this human population study were frequently MDR and harbored clinically relevant antimicrobial resistance genes. The findings demonstrate that CTX-M-15-and ESBL-variants of SHV-type (SHV-106 and SHV-27) (associated in two cases with carbapenemases) are the most frequent mechanisms of resistance in ESBL-producing *K*. *pneumoniae* isolates implicated in bacteremia in the tested hospital. Additionally, our study demonstrates the presence of high-risk international clones (ST11, ST15 and ST348) among these ESBL-producing *K*. *pneumoniae* isolates. More studies should be carried out in the future to track the evolution of these type of β-lactamases in different environments.

## Supporting information

S1 File (PDF)

S2 File (PDF)

S3 File (PDF)
