# Supplementary material for: Multidrug-resistant Klebsiella pneumoniae harboring extended spectrum β-lactamase encoding genes isolated from human septicemias
Source: PLoS One. 2021 May 4;16(5):e0250525. doi: 10.1371/journal.pone.0250525 (PMC8096088; doi:10.1371/journal.pone.0250525)
Supplement: S1 File — (PDF) [file pone.0250525.s001.pdf]

utad chtmad

PROTOCOLO DE COOPERAÇÃO

A Universidade de Trás-os-Montes e Alto Douro, através da investigadora responsável Prof. Doutora Patrícia Alexandra Curado Quintas Dinis Poeta do Departamento de Ciências Veterinárias, está empenhada em estudar os mecanismos de resistências a antibióticos em estirpes bacterianas (*Enterobactérias* resistentes aos beta-lactâmicos, *Enterococcus* spp., *klebsiella pneumoniae* resistente aos carbapnemos, *Acinetobacter baumannii*, *Staphylococcus* resistentes à meticilina e *Streptococcus* B) de origem hospitalar.

Neste contexto, a Universidade de Trás-os-Montes e Alto Douro e o Centro Hospitalar de Trás-os-Montes e Alto Douro, EPE, manifestam interesse recíproco em estreitar laços de cooperação, os quais visam o aproveitamento das potencialidades próprias para a realização de actividades comuns, de forma a valorizar os conhecimentos actuais nesta área da saúde pública.

Atendendo a que se trata de um projecto de enorme interesse para a saúde no âmbito local e nacional,

A UNIVERSIDADE DE TRÁS-OS-MONTES E ALTO DOURO, pessoa colectiva de direito público titular do número de identificação 501345361, com Sede na Quinta de Prados em Vila Real, abreviadamente designada por UTAD e aqui representada pela investigadora responsável Prof. Doutora Patrícia Alexandra Curado Quintas Dinis Poeta e

O CENTRO HOSPITALAR DE TRÁS-OS-MONTES E ALTO DOURO, EPE, titular do número de identificação 508100496, com Sede na Av. da Noruega em Vila Real, abreviadamente designada por CHTMAD e aqui representado pelo Dr. João Manuel Ferreira Gaspar, Director Clínico do Conselho de Administração

- Assinam o presente Convénio de Cooperação no domínio da investigação que se regerá pelas seguintes cláusulas:

1ª

As duas Instituições comprometem-se a conjugar esforços com vista ao desenvolvimento de cooperação no domínio da investigação técnica e científica.

2ª

Esta Cooperação não acarretará encargos financeiros para ambas as partes.

3ª

O papel do Centro Hospitalar de Trás-os-Montes e Alto Douro será o de conceder as estirpes bacterianas isoladas no laboratório de Análises Clínicas

4ª

A Universidade de Trás-os-Montes e Alto Douro, analisará todos os mecanismos de resistência aos antibióticos das estirpes cedidas

O presente protocolo é feito em dois exemplares, que vão ser assinados pelos representantes dos outorgantes, destinando-se um exemplar a cada um deles.

Vila Real, 23 de Janeiro de 2018

Investigadora Responsável

Profª Doutora Patricia Alexandra Curado Quintas Dinis Poeta

Pelo Conselho de Administração do Centro Hospitalar de Trás-os-Montes e Alto Douro

Dr. João Manuel Ferreira Gaspar
