# Supplementary material for: Multidrug-resistant Klebsiella pneumoniae harboring extended spectrum β-lactamase encoding genes isolated from human septicemias
Source: PLoS One. 2021 May 4;16(5):e0250525. doi: 10.1371/journal.pone.0250525 (PMC8096088; doi:10.1371/journal.pone.0250525)

UNIÃO EUROPEIA
Fundos Europeus Estruturais e de Investimento

PORTUGAL 2020

Identificação do Beneficiário

Nº de Identificação Fiscal	501345361
Designação Social	UNIVERSIDADE DE TRAS OS MONTES E ALTO DOURO
Morada (Sede Social)	AV ALMEIDA LUCENA 1
Localidade	VILA REAL
Código Postal	5000-000 VILA REAL
Distrito	Vila Real
Concelho	Vila Real
Telefone(s)	259350150
E-mail	baltazar@utad.pt
Telefax	259350480
URL	http://www.utad.pt
Data de Constituição	1979-09-14
Data de Início de Atividade	1979-09-14
Matriculada sob o Nº	501345361
Conservatória do Registo Comercial	
Natureza Jurídica	Pessoa Colectiva de Direito Público
Capital Social	€
CAE Principal	85420 - Ensino superior
Tipo de Entidade	Instituições de ensino superior, seus institutos e unidades de I&D
Entidade Não Empresarial do SI&I	Sim
Público/Privado	Público

Identificação dos códigos de validação da IES - Informação Empresarial Simplificada/Declaração anual dos 3 anos anteriores ao ano de candidatura

2015	
2014	
2013	

Identificação do Concurso

**Aviso:**	02/SAICT/2017
**Designação:**	Projetos de Desenvolvimento e Implementação de Infraestruturas de Investigação inseridas no RNIE
**Programa Operacional:**	Programa Operacional Regional do Norte
**Objetivo Temático:**	OT 1 - Reforçar a investigação, o desenvolvimento tecnológico e a inovação
**Prioridade de Investimento:**	PI 1.1 - O reforço da infraestrutura e das capacidades de investigação e inovação (I&I)
**Tipologia de Intervenção:**	TI 45 - Investigação científica e tecnológica
**Fundo:**	FEDER

Identificação do Projeto

**Nº do Projeto:**	030101
**Código da Operação:**	NORTE-01-0145-FEDER-030101

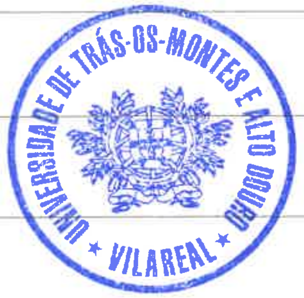

Supplement: S2 File — (PDF) [file pone.0250525.s002.pdf]
