# Supplementary material for: Multidrug-resistant Klebsiella pneumoniae harboring extended spectrum β-lactamase encoding genes isolated from human septicemias
Source: PLoS One. 2021 May 4;16(5):e0250525. doi: 10.1371/journal.pone.0250525 (PMC8096088; doi:10.1371/journal.pone.0250525)
Supplement: S3 File — (PDF) [file pone.0250525.s003.pdf]

ETHICS COMMITTEE

STATEMENT

Project name: CAREBIO2 - Comparative assessment of antimicrobial resistance in environmental biofilms through proteomics - towards innovative theranostic biomarkers

Dear Prof. Patrícia Poeta,

The Ethics Committee of University of Trás-os-Montes e Alto Douro (EC-UTAD) reviewed your application to obtain an ethical assessment for the above-mentioned project.

The following members of EC-UTAD participated in the ethical assessment:

Name	Qualification	Role of Ethics Committee	Gender	Affiliation to UTAD
Eurico Vasco F. Amorim	Professor	Member/President	M	Yes
Isabel O´Neill M. Gaivão	Professor	Member	F	Yes
José Albino G. Alves Dias	Professor	Member	M	Yes
Maria Cristina Q. Antunes	Professor	Member	F	Yes

This EC-UTAD is working following the Helsinki Declaration (ICH-GCP principles), compliance with Schedule Y / ICMR Guidelines, the Oviedo Convention, and other applicable regulation. None of the researchers participating in this study took part in the decision making and voting for this assessment.

Based on the review of Project documentation, the EC-UTAD states a unanimous favourable ethical opinion about the request as submitted.

The EC-UTAD expects to be informed about the progress of the study, any Serious Adverse Events occurring in the course of the study, any revision in the protocol, and requests an electronic copy of the final report.

UTAD, November 8, 2019

Assinado por : **EURICO VASCO FERREIRA AMORIM**
Num. de Identificação: BI087371847
Data: 2019.11.11 17:52:59 +0000

CARTÃO DE CIDADÃO

Eurico Vasco Ferreira Amorim

President of the Ethics Committee of UTAD

E-mail: comissaoetica@utad.pt

QUINTA DE PRADOS
5000-801 VILA REAL
Portugal
TEL. 259 350 000
FAX 259 350 480
